# Critical immunity gaps: Waning diphtheria protection among Yemen’s displaced populations calls for urgent booster strategies

**DOI:** 10.1016/j.ijregi.2026.100861

**Published:** 2026-02-14

**Authors:** Nazeh Al-Abd, Olawale Quazim Junaid, Abeer Ali Alausji, Abdulkhaleq Faiz Ben Laswed, Moataz Anwar Albehani, Sagir Mustapha, Omar Bamaga

**Affiliations:** 1 Department of Health Sciences, Faculty of Medicine and Health Sciences, University of Science and Technology, Aden, Yemen; 2 Department of Para-clinic, Faculty of Medicine and Health Sciences, University of Aden, Yemen; 3 Department of Biological Sciences, Faculty of Science, Federal University of Kashere, Gombe Satet, Nigeria; 4 Department of Parasitology, Faculty of Medicine, Universiti Malaya, Kuala Lumpur, Malaysia; 5 Department of Pharmaceutical Life Sciences, Faculty of Pharmacy, Universiti Malaya, Kuala Lumpur, Malaysia; 6 Department of Pharmacology and Therapeutics, Faculty of Pharmacy, Ahmadu Bello University, Zaria, Nigeria; 7 Department of Fundamental Medical Sciences, Faculty of Nursing, Hadhramout University, Hadramout, Yemen

**Keywords:** Diphtheria, Seroprevalence, Displaced population, Yemen, Humanitarian crisis, Vaccine immunity

## Abstract

**Objectives:**

This study evaluates seroprevalence of diphtheria toxoid immunoglobulin G antibodies among displaced populations in Abyan Governorate, which is to the best of our knowledge the first such assessment in Yemen’s conflict setting.

**Methods:**

A cross-sectional study in 390 displaced individuals (aged 1 month–90 years) assessed anti-diphtheria antibody levels through enzyme-linked immunosorbent assay. Geometric mean titers (GMTs) and seroprotection rates were analyzed by age, gender, and vaccination status.

**Results:**

Overall seroprotection was 76.2%, with 23.8% susceptible (<0.01 IU/ml). GMTs decreased significantly with age (1.04 IU/ml in those aged ≤10 years vs 0.78 IU/ml in those aged >40 years; *P* <0.05). Alarmingly, 63.8% of adults aged ≥40 years lacked protection. Although GMTs showed no gender difference (males: 1.04 ± 0.17; females: 1.05 ± 0.23 IU/ml), males had higher long-term protection (56.3% vs 44.0%, *P* <0.05). Individuals vaccinated exhibited better protection (56.0% vs 35.5%, *P* <0.05), yet 15.3% remained susceptible, suggesting cold-chain or dosing failures.

**Conclusions:**

Yemen’s displaced populations face critical immunity gaps, particularly among older adults. Suboptimal vaccine effectiveness and decreasing immunity underscore the need for: targeted booster campaigns for adults; strengthened vaccine delivery systems; and integrated serosurveillance in conflict zones. These findings provide evidence for revising national immunization strategies in humanitarian crises.

## Highlights

• First conflict-zone serosurvey in Yemen reveals critical protection gaps in adults aged >40 years.
• Age-dependent immunity reduction.
• Vaccination system failures.
• Male participants exhibit higher long-term protection despite similar geometric mean titers.
• Proposes targeted adult booster campaigns and strengthened serosurveillance in conflict zones.

## Introduction

Diphtheria is an acute bacterial infection with epidemic potential, primarily caused by toxigenic strains of *Corynebacterium diphtheriae*. In addition, two related species, *C. ulcerans* and *C. pseudotuberculosis*, can also produce diphtheria toxin and cause clinically similar disease [1]. The primary transmission routes for diphtheria include respiratory droplets, direct contact with infected secretions, or physical touch. Toxin-producing strains of *C. diphtheriae* are responsible for most cases of pharyngeal and nasopharyngeal infections, along with serious complications including myocarditis, peripheral neuropathy, and systemic toxicity. In contrast, infections caused by non-toxigenic strains typically produce milder clinical manifestations. Characteristic pathological features such as pseudomembrane formation on mucosal surfaces may lead to respiratory obstruction, particularly in cases of respiratory diphtheria [2]. Diphtheria has become an uncommon disease in some parts of the world owing to widespread immunization campaigns, but it remains endemic in others, including Eastern Europe, South America, Africa, and Southeast Asia.

Globally, a total of 7097 diphtheria cases were reported in 2019 with a high mortality rate of 5-10%, particularly among young children [3]. Nevertheless, recent reports of diphtheria cases in Yemen have prompted concerns about the potential resurgence of the disease in the country [4]. Diphtheria killed 122 children of 2055 suspected cases reported in 2023; thus, this implies a case fatality rate (CFR) of approximately 5.9% in Yemen [5]. Previously, from October 2017 to April 2020, there were more than 5700 probable diphtheria cases and 330 associated deaths reported in nearly all governorates of Yemen [6].

Diphtheria outbreaks have re-emerged globally in regions experiencing vaccination program disruptions, including conflict zones and areas with health care system collapse. Recent epidemiological reports document resurgences in Indonesia (2017), Venezuela (2019), Yemen (2017-2023), and among Rohingya refugee populations in Bangladesh (2018-2022) [[7], [8], [9], [10]]. Notably, Yemen’s 2017 outbreak yielded more than 2200 confirmed cases with an incidence rate of eight cases per 100,000 people, whereas there has been a significant increase of 57% diphtheria cases in 2023, thus exposing critical gaps in population immunity [4,11].

The diphtheria outbreak in Yemen has revealed a notable shift in the age distribution of cases compared with pre-conflict periods, primarily affecting older children and young adults, a pattern typical of populations with low or decreasing immunity [12]. Early in the outbreak of 2017, children younger than 20 years accounted for approximately 79% of suspected cases [12], whereas another study in Sada’a (2017-2020) found that children aged between 10 and 15 years constituted approximately 35% of the cases [13]. Notably, approximately 90% of fatalities were reported in children younger than 15 years during the 2017 outbreak [12,14] whereas higher CFRs have been consistently reported among the same age group [11]. Demographic analysis of cases reported at Hodeida Governorate revealed that diphtheria disproportionately affected females (63.1%) [15] whereas Shedaiwah et al. [4], reported that adult females were particularly affected, with poor vaccination and contact with cases identified as primary contributors in the Damt district of Al Dhalea Governorate.

The resurgence of diphtheria in Yemen has been linked to the dramatic decrease in vaccination coverage, particularly for the third dose of the diphtheria-tetanus-pertussis (DTP3) vaccine. The primary driver of the outbreak is the critically low DTP3 vaccination coverage, which plummeted to 46% in 2023 from 77% in 2012, leaving a massive and growing number of children unprotected and ensuring the persistence of the outbreak [16]. Furthermore, the proportion of children with zero dose (those who have not received any routine vaccinations) is projected to reach 28% in 2024 [16]. This immunity gap is most pronounced among internally displaced persons (IDPs), who face overcrowded living conditions and limited access to primary health care.

The resurgence mechanisms appear multifactorial: introduction of novel toxigenic *C. diphtheriae* biotypes; suboptimal pediatric vaccination coverage; and decreasing adult immunity without booster programs [17,18]. Compounding factors include overcrowded living conditions and inadequate hygiene infrastructure, which facilitate respiratory transmission, so it is not uncommon that the resurgence of this disease can be found in crisis-driven settings [2].

The body’s defense against diphtheria, known as humoral immunity, is primarily driven by the production of immunoglobulin G (IgG) antibodies targeting the diphtheria toxin. These antibodies can develop through natural infection, passive transfer (such as maternal antibodies), or active immunization (through vaccines). However, as diphtheria cases have decreased significantly, natural exposure to the bacteria, and thus the chance to build or reinforce immunity, has also greatly decreased [19].

This study seeks to evaluate the current levels of diphtheria immunity among IDPs residing in the Al-Tomaisi camp. The findings are expected to offer insights into the immune protection against diphtheria within a Yemeni population. In addition, the results could help inform potential adjustments to vaccination strategies, such as revising the recommended age for initiating immunization or expanding diphtheria vaccine coverage to other age groups. Yemen is one of the under-resourced countries that have experienced civil war since March 2015 that ultimately led to the destruction of the infrastructure of the country, including health services, and caused a severe lack of personnel, medication, and equipment, and the degradation of essential services. However, the conflict forces large populations to leave their homes searching for safe shelter.

Assessing protective herd immunity requires systematic serosurveillance to identify immunity gaps [3] and targeted vaccination campaigns prioritizing high-risk groups, particularly displaced communities in which transmission risks are amplified by overcrowding and limited health care access [2]. Hence, strengthening immunization programs in these vulnerable populations is now imperative to prevent nationwide diphtheria spread.

## Materials and methods

A cross-sectional community study was performed over 4 months (March to June 2021) involving IDPs living in the Al-Tomaisi camp in Zinjibar City, Abyan. This camp houses approximately 200 families, and it lies in approximately 13°08′10′'N latitude and 45°22′27′'E longitude. Zinjibar City, the capital of Abyan governorate, is situated on the southern coast of Yemen. Displaced people are those compelled to abandon their native locales without crossing an internationally recognized state boundary.

Owing to security constraints, the absence of a reliable population register, and the informal nature of the IDP settlement, a probability-based random sampling method was not feasible. A convenience sampling approach was used to recruit participants on the basis of voluntary participation. The research team established a data collection station at a central location within the camp. Community health workers and camp leaders informed residents of the study. All individuals presenting to the station who met the inclusion criteria (e.g., healthy individual, residency in the camp, no prior confirmed diphtheria diagnosis) and were willing to give informed consent and to participate were enrolled.

### Sample size

Sample size for this study was calculated using the formula for estimating minimal sample size provided by Naing et al. [20], using a 95% confidence interval. According to Mohammed et al. [21], the prevalence of protective anti-diphtheria immunity in Saudi Arabia was 31.7%. On the basis of this estimate, the minimum required sample size was determined to be 332. The sample size calculation used the formula:

$$n=\frac{Z^{2}P\left(1-P\right)}{d^{2}}$$

n = sample size,
Z = Z statistic for the desired confidence level (1.96 for 95% confidence),
P = anticipated prevalence, and
d = margin of error.

### Data and sample collection

A structured questionnaire was used to collect data on sociodemographic factors, including age, gender, vaccination history, and prior diphtheria infection. Moreover, anti-diphtheria antibody levels were measured among IDPs residing in the Al-Tomaisi camp, Abyan governorate, Yemen. Written informed consent was obtained from all participants.

Blood samples (approximately 5 ml) were aseptically collected from all participants through venipuncture. Each sample was placed in a gel tube, properly labeled with the participant's name, collection date, and a unique identification number. After allowing the blood to clot, samples were centrifuged at 3000 rpm for 5 minutes. The resulting serum was carefully transferred into pre-labeled Eppendorf tubes using a Pasteur pipette and stored at –20°C until further analysis.

### Laboratory analysis

All laboratory analyses were performed at the University of Aden’s Faculty of Medicine and Health Sciences. Serum diphtheria IgG levels were quantified using an enzyme-linked immunosorbent assay (ELISA) with the ELISA Classic Diphtheria IgG® kit (Demeditec Diagnostics GmbH, Kiel, Germany), following the manufacturer’s instructions. This quantitative immunoassay uses diphtheria-specific antigen-coated microwells. Test samples were diluted and incubated in the wells, allowing specific IgG antibodies to bind to the immobilized antigen. After a washing step to remove unbound components, an enzyme conjugate was added to form antibody-antigen complexes. After another wash, a chromogenic substrate was introduced, producing a colorimetric reaction proportional to the IgG antibody concentration. The reaction was terminated at a predetermined timepoint, and absorbance values were measured using a microwell plate reader. Results were interpreted by comparing sample readings with parallel-run calibrators and controls.

Diphtheria immunity status was categorized according to World Health Organization (WHO) [22] criteria based on serum anti-diphtheria antibody levels (IU/ml): <0.01 IU/ml: no protection (susceptible); 0.01-0.09 IU/ml: some degree of protection; and ≥0.1 IU/ml: long-term protection.

### Statistical method

Data analysis was performed using SPSS software (version 24.0). Quantitative variables were summarized as means ± standard deviations (SD), frequencies, and percentages. Geometric mean titers (GMTs) were derived by log-transforming antibody titers and computing the antilog of their mean values. The prevalence of different immunity levels among displaced individuals was reported as percentages.

Associations between sociodemographic factors and diphtheria protection levels were evaluated using chi-square tests. A significance threshold of *P* <0.05 was adopted for all statistical analyses.

## Results

### Demographic characteristics of the study population

A total of 390 displaced individuals voluntarily participated in this study. The cohort comprised 174 males (44.6%) and 216 females (55.4%). Participants’ ages ranged from 1 month to 90 years, stratified into five age groups: 0-9 years, 10-19 years, 20-29 years, 30-39 years, and ≥40 years. The mean age of participants was 19.8 ± 16.5 years, with the largest proportion (41.0%, n = 160) belonging to the 0–9-year age group, followed by the 10–19-year age group (20.8%, n = 81) (Table 1).

**Table 1:** Demographic and seroprotection profile of displaced individuals assessed for diphtheria immunity.

Characteristic	Category	Frequency (n)	Percentage (%)
**Sex**	Male	174	44.6
	Female	216	55.4
**Age group (years)**	0-9	160	41.0
	10-19	81	20.8
	20-29	56	14.4
	30-39	46	11.8
	≥40	47	12.1
**Seroprotection status**	Long-term protection (≥0.1 IU/ml)	193	49.5
	Some degree of protection (0.01-0.09 IU/ml)	104	26.7
	No protection (<0.01 IU/ml)	93	23.8
	**Total**	**390**	**100.0**

### Seroprevalence of anti-diphtheria toxoid IgG antibodies

The GMT of anti-diphtheria toxoid IgG antibodies across the study population was 1.05 ± 0.20 IU/ml. Serological analysis revealed that 23.8% of participants (n = 93) had antibody levels lower than 0.01 IU/ml, indicating susceptibility to diphtheria. Some degree of protection was observed in 26.7% of participants (n = 104), whereas 49.5% (n = 193) exhibited long-term protection (Table 1).

GMTs of anti-diphtheria toxoid IgG antibodies were comparable in males (1.04 ± 0.17 IU/ml) and females (1.05 ± 0.23 IU/ml). However, stratification by immunity categories revealed significant gender-based disparities (*P* <0.05). Among males, 98 (56.3%) showed long-term protection; 49 (28.2%) showed some degree of protection, and 27 (15.5%) were seronegative. In contrast, females exhibited lower long-term protection (44.0%, n = 95) and higher susceptibility (30.6%, n = 66) (Table 2).

**Table 2:** Seroprotection against diphtheria in displaced individuals in relation to gender.

Gender	No. examined	Geometric mean titer (IU/ml) Mean ± SD	Immunity category			*P*-value (Immunity category)
			Long-term protection	Some degree of protection	No protection	
			≥0.1 IU/ml (%)	0.0 1-0.09 IU/ml (%)	<0.01 IU/ml (%)	
Male	174	1.04 ± 0.17	98 (56.3)	49 (28.2)	27 (15.5)	0.002
Female	216	1.05 ± 0.23	95 (44.0)	55 (25.4)	66 (30.6)	
**Total no.**	**390**		**193 (49.5)**	**104 (26.7)**	**93 (23.8)**	

**Figure 1:**
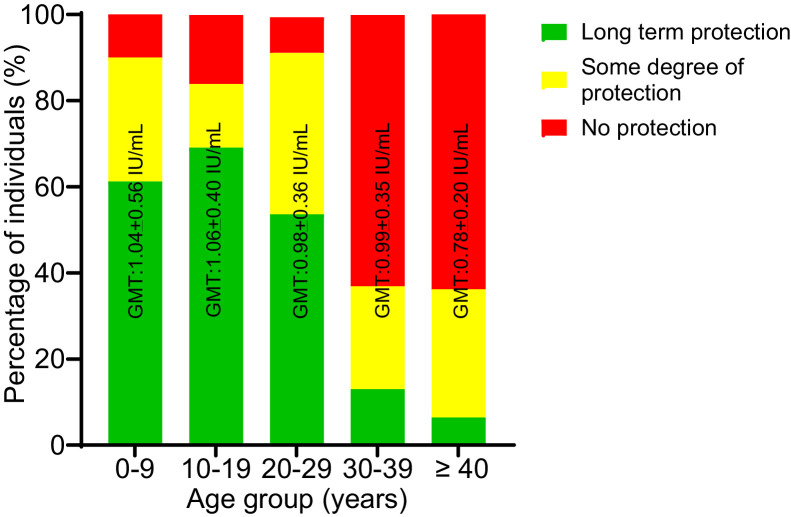
Seroprotection against diphtheria in displaced individuals by age group. Number of displaced individuals examined for the age groups 0-9, 10-19, 20-29, 30-39 and ≥40 years were 160, 81, 56, 46 and 47, respectively.

GMTs did not differ significantly across age groups, although variations in immunity categories were notable. The highest proportion of long-term protection was observed in the 0–9-year (61.3%) and 10–19-year (69.1%) age groups. Conversely, seropositivity decreased markedly in older cohorts, with only three individuals (6.4%) aged ≥40 years exhibiting long-term protection. Susceptibility increased with age while showing a peak among those in the ≥40-year group (Figure 1).

GMT, geometric mean titer ± standard deviation.

### Impact of vaccination status on seroprotection

No significant difference in GMTs was observed between individuals vaccinated (1.04 ± 0.67 IU/ml) and unvaccinated (1.05 ± 0.53 IU/ml). However, vaccination status significantly influenced immunity categories (*P* <0.05). Among vaccinated participants, 149 (56.0%) had long-term protection, compared with 44 of unvaccinated individuals (35.5%). Susceptibility was markedly higher in the unvaccinated (42.7%, n = 53) than in the vaccinated cohort (15.0%, n = 40) (Table 3).

**Table 3:** Seroprotection by vaccination status of displaced individuals.

Vaccination status	No. examined	Geometric mean titer (IU/ml) Mean ± SD	Immunity category			*P*-value (Immunity category)
			Long-term protection ≥0.1 IU/ml (%)	Some degree of protection ≥0.01-0.091 IU/ml (%)	No protection <0.01 IU/ml (%)	
Vaccinated	266	1.04 ± 0.67	149 (56.0)	77 (28.9)	40 (15.0)	0.001
Unvaccinated	124	1.05 ± 0.53	44 (35.5)	27 (21.8)	53 (42.7)	
**Total**	**390**		**193 (62.3)**	**104 (26.7)**	**93 (23.8)**	

### Immune response stratified by gender, age, and vaccination status

Susceptibility rates were higher in males (28.7%, n = 50) than in females (20.0%, n = 43) (*P* <0.05). Notably, all vaccinated males were immune, whereas 50 susceptible males (100.0%) were unvaccinated. Among females, 98 vaccinated individuals (86.0%) were immune, whereas 27 susceptible females (26.5%) were unvaccinated. Vaccination history significantly predicted immune status (*P* <0.05) (Tables 4).

**Table 4:** Immune protection against diphtheria by gender and vaccination status of displaced individuals.

Variable	Category	Protected, n (%)	Susceptible, n (%)	Total, n (%)	P-value
**Gender**	Male	124 (71.3)	50 (28.7)	174 (100.0)	**0.028**
	Female	173 (80.0)	43 (20.0)	216 (100.0)	
**Vaccination**	Vaccinated	226 (85.0)	40 (15.0)	266 (100.0)	**0.001**
	Unvaccinated	71 (57.3)	53 (42.7)	124 (100.0)	
**Gender + Vaccination**	Male-vaccinated	124 (100)	0 (0)	124 (100.0)	**0.001**
	Male-unvaccinated	0 (0)	50 (100)	50 (100.0)	
	Female-vaccinated	98 (86.0)	16 (14.0)	114 (100.0)	
	Female-unvaccinated	75 (73.5)	27 (26.5)	102 (100.0)	

The highest seroprotection was observed in the youngest age group (0-9 years), with 83.1% protected. Within this group, vaccination status had a profound impact; 110 vaccinated children (98.2%) were protected, compared with only 23 of the unvaccinated children (47.9%). Protection rates generally decreased with increasing age. Among those aged 10-19 and 20-29 years, overall seroprotection was 86.4% and 78.6%, respectively, with vaccinated subgroups maintaining higher protection (90.0% and 80.0%) than their unvaccinated peers (76.2% and 75.5%) (Table 5).

**Table 5:** Immune protection against diphtheria by age group and vaccination status of displaced individuals.

Age group (years)	Vaccination status	Total examined (n)	Protected n (%)	Susceptible n (%)
0-9	Vaccinated	112	110 (98.2)	2 (1.8)
	Unvaccinated	48	23 (47.9)	25 (52.1)
	**Total**	**160**	**133 (83.1)**	**27 (16.9)**
10-19	Vaccinated	60	54 (90.0)	6 (10.0)
	Unvaccinated	21	16 (76.2)	5 (23.8)
	**Total**	**81**	**70 (86.4)**	**11 (13.6)**
20-29	Vaccinated	40	32 (80.0)	8 (20.0)
	Unvaccinated	16	12 (75.5)	4 (25.5)
	**Total**	**56**	**44 (78.6)**	**12 (21.4)**
30-39	Vaccinated	27	18 (66.7)	9 (33.3)
	Unvaccinated	19	11 (57.9)	8 (42.1)
	**Total**	**46**	**29 (63.0)**	**17 (37.0)**
≥40	Vaccinated	27	12 (44.4)	15 (55.6)
	Unvaccinated	20	9 (45.0)	11 (55.0)
	**Total**	**47**	**21(44.7)**	**26 (55.3)**
Overall	Vaccinated	266	226 (85.0)	40 (15.0)
	Unvaccinated	124	71 (57.3)	53 (42.7)
	**Grand Total**	**390**	**297 (76.1)**	**93 (23.9)**

A notable reduction in immunity was observed in older age groups. For individuals aged 30-39 and ≥40 years, overall protection was 63.0% and 44.7%, respectively. In the ≥40-years group, there was no significant difference in protection between vaccinated (44.4%) and unvaccinated (45.0%) individuals, suggesting decreasing vaccine-induced immunity in the absence of natural exposure or booster doses (Table 5).

## Discussion

This study reveals notable differences in immunity related to age, gender, and vaccination status, offering crucial insights into the seroprevalence of diphtheria toxoid IgG antibodies among Yemeni displaced communities. Yemen’s protracted humanitarian crisis, marked by political instability and a collapsed health system, has severely disrupted routine immunization programs, leading to resurgent diphtheria outbreaks despite historically widespread vaccine coverage [23]. Our study, which is to the best of our knowledge the first to assess diphtheria seroprevalence among Yemen’s displaced populations, reveals alarming immunity gaps: 23.8% of participants were seronegative, and protection decreased dramatically with age, with 63.8% of adults older than 40 years lacking protection. Crucially, even vaccinated individuals showed unexpected susceptibility (15.3%), suggesting systemic issues such as cold-chain failures, suboptimal dosing, or inadequate booster coverage.

Nearly 24% of participants lacked protective antibody titers (<0.01 IU/ml), highlighting susceptibility to diphtheria outbreaks in displaced communities. This aligns with studies in similar crisis-affected populations, in whom overcrowding and limited health care access reduce vaccine coverage [2]. The highest susceptibility was observed in adults older than 40 years (63.8%), consistent with global data showing decreasing immunity in older age groups [24]. Notably, diphtheria-containing vaccines (DTP) were introduced into Yemen’s Expanded Programme on Immunization in the late 1970s, with gradual expansion in coverage over subsequent decades [16]. Children aged 0–9 years in the present study are more likely to have benefited from routine immunization services or supplementary campaigns before and during the early years of conflict, which explains their relatively higher seroprotection.

Data on diphtheria immunity among displaced populations remain scarce, necessitating comparisons with general populations in other countries. In this study, the seroprotection rate was 76.2%, lower than rates reported in Thailand (90.9%) during a non-outbreak period [25] but comparable to studies from Austrian (64.0%), Saudi (68.3%) and Greek (68.5%) populations [21,26,27]. In contrast, our findings exceeded seroprotection rates reported from Malaysia (43.3%) and Belgium (29.0%) [28,29], where studies were conducted in endemic-free settings. These disparities may reflect contextual differences, particularly the timing of studies relative to outbreaks (unlike Thailand and our study, post-outbreak); Malaysia, Greece, and Belgium were assessed during endemic stability.

Females exhibited lower long-term protection (44.0% vs 56.3% in males) and higher susceptibility (30.6% vs 15.5%), contrasting with studies reporting no gender-based differences in high-income settings [30]. According to Sameer et al. [4], women (especially those of reproductive age) exhibit a greater susceptibility to diphtheria, even though they should theoretically be protected by tetanus-diphtheria vaccination. This disparity points to broader gender-based inequities in health care access, exacerbated by cultural restrictions, reduced mobility, and the persistent conflict in Yemen. The findings emphasize the urgent need for tailored public health strategies that specifically address the barriers preventing women from receiving adequate medical care.

Despite Yemen's national immunization program recommending three doses of DTP-hepatitis B vaccine at 2, 4, and 18 months of age followed by a tetanus-diphtheria booster at 6 years, our findings reveal concerning gaps in population immunity against diphtheria. This study indicates a clear age-dependent reduction in protective antibody levels, with older displaced persons showing significantly lower mean anti-diphtheria titers than younger individuals and reduced protection [24]. This pattern aligns with global observations of decreasing immunity in older age groups [3].

Vaccinated individuals had significantly higher long-term protection (56.0% vs 35.5%), reinforcing the importance of routine immunization. However, 15.3% of vaccinated participants remained susceptible, suggesting potential cold-chain breaches, suboptimal dosing, or genetic factors affecting vaccine response [17].

This finding, that vaccinated males exhibit a higher proportion of seroprotection against diphtheria than do vaccinated females, highlights a significant gender-based immunity gap. However, this factor may be partially explained by general gender-specific differences in immune response. Other studies have reported that adult women are more likely to lack seroprotection against diphtheria than are men [31,32]. The most compelling hypothesis could be related to the cumulative effect of maternal vaccination and the potential for blunted booster responses or temporary antibody depletion due to placental transfer [33,34].

In contrast, a notable finding from our study shows that a substantial proportion of unvaccinated women (73.5%) had protective antibody levels, whereas all unvaccinated men were serologically susceptible. This observation is biologically plausible and has been described in other settings, where women experience increased opportunities for natural exposure through caregiving roles, household contact with children, and care of sick individuals, causing natural immune boosting. Historically, Yemen’s maternal immunization program has relied primarily on tetanus toxoid (TT) vaccination to prevent neonatal tetanus [12]. TT does not confer protection against diphtheria; therefore, maternal vaccination cannot explain diphtheria seroprotection observed among women in this study. The combined tetanus–diphtheria vaccine was introduced more recently and inconsistently, with implementation significantly disrupted by ongoing conflict [35].

In addition, socio-cultural barriers to health care access in a conflict-affected setting such as the study area could play a major role. Although the data focus on vaccinated individuals, the quality and timing of that vaccination are critical. Women in displaced communities may face greater challenges in accessing routine or booster vaccination services owing to mobility restrictions, safety concerns, or prioritization of other family needs. Furthermore, poor documentation of vaccination history, a common issue in humanitarian crises, makes it difficult to ascertain whether the “vaccinated” status is based on a complete and timely primary series and appropriate boosters.

The reduced seroprotection among adults older than 30 years may result from multiple factors: suboptimal immune responses to childhood vaccination, decreased natural boosting due to improved hygiene, and potential gaps in historical vaccine coverage during periods of conflict [17,23]. These findings emphasize the need for targeted booster vaccination strategies, particularly for older populations in humanitarian settings.

Our data consistently affirm the fundamental role of vaccination in conferring protection against diphtheria. Across all age groups, vaccinated individuals maintain a significantly higher seroprotection rate than do their unvaccinated counterparts. However, the most salient finding is the clear, inverse relationship between age and seroprotection among the vaccinated population. The half-life of protective diphtheria antitoxin antibodies is estimated to be approximately 10 years, meaning that individuals who received their last booster in childhood or adolescence are likely to become susceptible in adulthood without subsequent booster doses [36].

Yemen’s health system must urgently reassess population immunity against diphtheria, given our findings challenge previous assumptions about herd immunity thresholds. Although it was suggested that 85% population immunity is required to prevent outbreaks [3], the WHO has emphasized that more than 90% coverage in children and more than 75% in adults are required to sustain herd protection in a population [35]. Alarmingly, 63% of displaced persons older than 30 years in our study lacked protective immunity, likely owing to insufficient booster vaccinations and reduced natural exposure to *C. diphtheriae*, factors that increase epidemic risks in humanitarian settings [23]. Therefore, our findings underscore the urgent need for targeted immunization strategies in humanitarian settings, when disrupted health care systems exacerbate vaccine-preventable disease risks.

Outbreaks of diphtheria in similar humanitarian crises, such as among the Rohingya refugees in Bangladesh, have been fueled by such immunity gaps across multiple age groups [9]. Our findings suggest that vaccination strategies in emergency settings must look beyond young children. The WHO [12] recommends mass vaccination campaigns with diphtheria toxoid-containing vaccines for all individuals in outbreak scenarios, regardless of age, if vaccine supply permits. This study supports expanding such preventive campaigns to include older children, adolescents, and adults in displaced populations, even in non-outbreak settings, to build herd immunity and prevent resurgence.

A limitation of this study is its cross-sectional design, which captures serostatus at a single timepoint and cannot definitively attribute the low titers in older adults solely to decreasing immunity vs primary vaccine failure or lack of vaccination. Furthermore, the exact vaccination history (number of doses, timing) was likely based on recall, which may be unreliable in displaced contexts. Another limitation is that serological assessment captures humoral immunity but does not account for cell-mediated immune responses.

## Conclusion

This study highlights critical gaps in diphtheria immunity among displaced populations in Yemen, with nearly one-quarter of individuals lacking protective antibody levels. The findings reveal age and gender disparities, with older adults (≥40 years) and females exhibiting higher susceptibility, alongside incomplete protection among vaccinated individuals. These results underscore the urgent need for targeted vaccination campaigns, improved surveillance, and gender-sensitive health care delivery in humanitarian settings.

Given the resurgence of diphtheria in the study area, our data support the implementation of booster doses for older age groups in the Al-Tomaisi camp and strengthened cold-chain systems to ensure vaccine efficacy. Future research should explore long-term immunity trends and barriers to health care access in displaced populations. Addressing these gaps is essential to preventing outbreaks and achieving global diphtheria elimination targets.

## Declaration of competing interest

The authors have no competing interests to declare.

## References

[1] Hanvatananukul P., Prasarakee C., Sarachai S., Aurpibul L., Sintupat K., Khampan R. (2020). Seroprevalence of antibodies against diphtheria, tetanus, and pertussis among healthy Thai adolescents. Int J Infect Dis.

[2] Polonsky J.A., Ivey M., Mazhar M.K.A., Rahman Z., le Polain de Waroux O., Karo B. (2021). Epidemiological, clinical, and public health response characteristics of a large outbreak of diphtheria among the Rohingya population in Cox’s Bazar, Bangladesh, 2017 to 2019: a retrospective study. PLOS Med.

[3] Truelove S.A., Keegan L.T., Moss W.J., Chaisson L.H., Macher E., Azman A.S. (2020). Clinical and epidemiological aspects of diphtheria: a systematic review and pooled analysis. Clin Infect Dis.

[4] Shedaiwah S., Alsharabi H., Anam L., Al Amad M.A. (2024). Risk factors of diphtheria outbreak in damt district of Al Dhalea Governorate, 2023 -Yemen: a case–control study. BMC Infect Dis.

[5] UNITED NATIONS CHILDREN’S FUND (2024).

[6] World Health Organization (2017).

[7] Harapan H., Anwar S., Dimiati H., Hayati Z., Mudatsir M. (2019). Diphtheria outbreak in Indonesia, 2017: an outbreak of an ancient and vaccine-preventable disease in the third millennium. Clin Epidemiol Glob Health.

[8] Badell E., Alharazi A., Criscuolo A., Almoayed K.A.A., Lefrancq N., Bouchez V. (2021). Ongoing diphtheria outbreak in Yemen: a cross-sectional and genomic epidemiology study. Lancet Microbe.

[9] Islam Z., Ahmed S., Rahman M.M., Karim M.F., Amin MR. (2022). Global stability analysis and parameter estimation for a diphtheria model: a case study of an epidemic in Rohingya refugee camp in Bangladesh. Comput Math Methods Med.

[10] Paniz-Mondolfi A.E., Tami A., Grillet M.E., Márquez M., Hernández-Villena J., Escalona-Rodríguez M.A. (2019). Resurgence of vaccine-preventable diseases in Venezuela as a regional public health threat in the Americas. Emerg Infect Dis.

[11] Moghalles S.A., Aboasba B.A., Alamad M.A., Khader YS. (2021). Epidemiology of diphtheria in yemen, 2017–2018: surveillance data analysis. JMIR Public Health Surveill.

[12] World Health Organization (2017).

[13] Al-Dar A.A., Al-Qassimi M., Ezzadeen F.H., Qassime M., Al Murtadha A.M., Ghaleb Y. (2022). Diphtheria resurgence in Sada'a-Yemen, 2017–2020. BMC Infect Dis.

[14] ACAPS (2017).

[15] Moulhee N.M.S., Alwesabi S.A.M., Taha M.S., Majam ASM. (2025). Epidemiological analysis of diphtheria cases in Hodeida, Yemen: a review of admission records from 2018 to 2022. J Med Pharm Sci (JMPS).

[16] World Health Organization (2024).

[17] Osarenren J., Omosigho P.O., Okesanya OJ. (2024). Global strategies for addressing diphtheria resurgence epidemiology clinical impact and prevention. Discovery Public Health.

[18] Vieira V.V., Ramos J.N., dos Santos L.S., Mattos-Guaraldi AL., de Filippis I. (2022). Molecular typing in bacterial infections.

[19] Gabutti G., Azzari C., Bonanni P., Prato R., Tozzi A.E., Zanetti A. (2015). Pertussis. Hum Vaccin Immunother.

[20] Naing L., Nordin R.B., Abdul Rahman H., Naing Y.T. (2022). Sample size calculation for prevalence studies using Scalex and ScalaR calculators. BMC Med Res Methodol.

[21] Mohammed A.R., Redwan E.M., Almehdar HA. (2017). Status of diphtheria immunity among Saudi population. J Pure Appl Microbiol.

[22] World Health Organization (2006).

[23] Dureab F., Al-Sakkaf M., Ismail O., Kuunibe N., Krisam J., Müller O. (2019). Diphtheria outbreak in Yemen: the impact of conflict on a fragile health system. Confl Health.

[24] Doherty T.M., Weinberger B., Didierlaurent A., Lambert P-H. (2025). Age-related changes in the immune system and challenges for the development of age-specific vaccines. Ann Med.

[25] Wanlapakorn N., Yoocharoen P., Tharmaphornpilas P., Theamboonlers A., Poovorawan Y. (2014). Diphtheria outbreak in Thailand, 2012; seroprevalence of diphtheria antibodies among Thai adults and its implications for immunization programs. Southeast Asian J Trop Med Public Health.

[26] Wagner A., Jasinska J., Schmid D., Kundi M., Wiedermann U. (2023). Lack of seroprotection against diphtheria in the Austrian population, in light of reported diphtheria cases in Europe, 2022. Euro Surveill.

[27] Papagiannis D., Thireos E., Mariolis A., Katsioulis A., Lampropoulos I.C., Tsiaousi I. (2024). Diphtheria and tetanus immunity status among Greek adults: results from a nationwide seroprevalence study. Vaccines (Basel).

[28] Yusoff A.F., Mohd Sharani Z.Z., Kee C.C., Md Iderus N.H., Md Zamri A.S.S., Nagalingam T. (2021). Seroprevalence of diphtheria toxoid IgG antibodies in the Malaysian population. BMC Infect Dis.

[29] Boey L., Bosmans E., Ferreira L.B., Heyvaert N., Nelen M., Smans L. (2021). Seroprevalence of antibodies against diphtheria, tetanus and pertussis in adult at-risk patients. Vaccines (Basel).

[30] Clarke KE. (2017).

[31] Völzke H., Kloker K.M., Kramer A., Guertler L., Dören M., Baumeister S.E. (2006). Susceptibility to diphtheria in adults: prevalence and relationship to gender and social variables. Clin Microbiol Infect.

[32] Fink A.L., Klein SL. (2015). Sex and gender impact immune responses to vaccines among the elderly. Physiology (Bethesda).

[33] Knuutila A., Barkoff A-M, Ivaska L., Tenhu E., Teräsjärvi J., van Gageldonk P. (2023). Effect of immunization during pregnancy and pre-existing immunity on diphtheria-tetanus-acellular pertussis vaccine responses in infants. Emerg Microbes Infect.

[34] Abu-Raya B., Giles M.L., Kollmann T. (2025). Co-administration of vaccines in pregnancy: unique challenges and knowledge gaps. Vaccine.

[35] World Health Organization (2016).

[36] Slifka M.K., Amanna IJ. (2019). Role of multivalency and antigenic threshold in generating protective antibody responses. Front Immunol.

